# Oestrogen‐activated autophagy has a negative effect on the anti‐osteoclastogenic function of oestrogen

**DOI:** 10.1111/cpr.12789

**Published:** 2020-03-11

**Authors:** Liang Cheng, Yunrong Zhu, Denghui Xie, Dianshan Ke

**Affiliations:** ^1^ Guangdong Provincial Key Laboratory of Bone and Joint Degeneration Diseases The Third Affiliated Hospital of Southern Medical University Guangzhou China; ^2^ Department of Orthopedics The Affiliated Jiangyin Hospital of Medical College of Southeast University Jiangyin China

**Keywords:** autophagy, LC3, oestrogen, osteoclastogenesis, RANKL

## Abstract

**Objectives:**

Oestrogen is known to inhibit osteoclastogenesis, and numerous studies have identified it as an autophagic activator. To date, the role of oestrogen in the autophagy of osteoclast precursors (OCPs) during osteoclastogenesis remains unclear. This study aimed to determine the effect of autophagy regulated by the biologically active form of oestrogen (17β‐estradiol) on osteoclastogenesis.

**Materials and methods:**

After treatment with 17β‐estradiol in OCPs (from bone marrow‐derived macrophages, BMMs) and ovariectomy (OVX) mice, we measured the effect of 17β‐estradiol on the autophagy of OCPs in vitro and in vivo. In addition, we studied the role of autophagy in the OCP proliferation, osteoclast differentiation and bone loss regulated by 17β‐estradiol using autophagic inhibitor or knock‐down of autophagic genes.

**Results:**

The results showed that direct administration of 17β‐estradiol enhanced the autophagic response of OCPs. Interestingly, 17β‐estradiol inhibited the stimulatory effect of receptor activator of nuclear factor‐κB ligand (RANKL) on the autophagy and osteoclastogenesis of OCPs. Moreover, 17β‐estradiol inhibited the downstream signalling of RANKL. Autophagic suppression by pharmacological inhibitors or gene silencing enhanced the inhibitory effect of 17β‐estradiol on osteoclastogenesis. In vivo assays showed that the autophagic inhibitor 3‐MA not only inhibited the autophagic activity of the OCPs in the trabecular bone of OVX mice but also enhanced the ability of 17β‐estradiol to ameliorate bone loss.

**Conclusions:**

In conclusion, our study showed that oestrogen directly enhanced the autophagy of OCPs, which inhibited its anti‐osteoclastogenic effect. Drugs based on autophagic inhibition may enhance the efficacy of oestrogen on osteoporosis.

## Graphical abstract

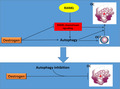

## 1 INTRODUCTION

The micro‐damage remodelling cycle is needed to maintain skeleton homeostasis under stress, and osteoclast plays a significant role in the above process due to its function to absorb bone matrix.^1^, ^2^ However, an excess of osteoclast number or activity causes bone mass reduction, eventually resulting in osteoporosis.^1^

Oestrogen, an endogenous hormone, can orchestrate bone remodelling. The lack of oestrogen is associated with increased bone remodelling rates and accelerated bone loss. Oestrogen replacement therapy has been reported to have a curative effect on osteoporosis. Oestrogen was found to increase the bone mineral density (BMD)^3^, ^4^, ^5^ and decrease the risk for fracture in post‐menopausal women.^6^, ^7^, ^8^ Another study showed that oestrogen effectively ameliorated the inhibition of bone histomorphological parameters caused by oestrogen deficiency.^9^, ^10^, ^11^ Similar results were found in other studies.^12^, ^13^, ^14^ However, conclusions have not been reached regarding the safety of oestrogen replacement therapy. The administration of oestrogen is accompanied by an increased risk of stroke, thromboembolism, coronary heart disease and breast cancer.^13^, ^14^, ^15^ Therefore, it is necessary to improve the therapeutic strategies utilizing oestrogen to treat osteoporosis to increase its efficacy and safety.

The inhibitory effect of oestrogen on osteoclastogenesis strongly contributes to its role in treating osteoporosis. Previous studies have shown that oestrogen inhibits osteoclast formation^16^, ^17^, ^18^ and increases osteoclast apoptosis.^19^, ^20^ However, the intrinsic mechanism underlying oestrogen‐regulated osteoclastogenesis has not been fully clarified.

Autophagy, as a protective mechanism at the cellular level, can enhance osteoclast formation and bone resorption activity.^21^, ^22^, ^23^, ^24^ Interestingly, numerous studies have suggested that oestrogen functions as an autophagic activator,^25^, ^26^, ^27^, ^28^, ^29^, ^30^, ^31^, ^32^ which might counteract the anti‐osteoclastogenic effect of this agent. Although the regulatory role of autophagy in bone loss caused by oestrogen withdrawal has been reported in many studies,^23^, ^24^, ^33^, ^34^, ^35^, ^36^, ^37^ the role of oestrogen‐mediated autophagy in osteoclastogenesis is still unknown. Therefore, the correlation between autophagy and oestrogen‐regulated osteoclastogenesis deserves further investigation.

The present results indicated that the presence of receptor activator of nuclear factor‐κB ligand (RANKL) influenced the development of autophagy in osteoclast precursors (OCPs), as 17β‐estradiol promoted autophagy in the absence of RANKL and inhibited RANKL‐enhanced autophagy. Mechanistically, 17β‐estradiol attenuated RANKL‐RANK‐TRAF6 (downstream signal molecule of RANKL) signalling. Furthermore, autophagic inhibition augmented the inhibitory effect of 17β‐estradiol on osteoclastogenesis and its protective effect against bone loss. Thus, our data provide the first evidence showing that oestrogen can affect osteoclastogenesis and bone integrity through the autophagic response.

## 2 MATERIALS AND METHODS

### 2.1 Animals

Four‐to‐eight‐week‐old C57BL/6 female mice (20‐25 g) were obtained from the Model Animal Research Center of Nanjing University (Nanjing, China). The experimental protocols were approved by the Institutional Animal Care and Use Committee of The Third Affiliated Hospital, Southern Medical University. The animals were housed in a common environment in which the room temperature was 20‐30°C and the humidity was 60%‐80%, and were fed a general laboratory diet.

### 2.2 Extraction and induction of OCPs

The tibiae from 4 to 8‐week‐old C57BL/6 mice were flushed with α‐MEM without serum. The bone marrow cells were incubated with α‐MEM containing 10% FBS, penicillin (100 U/mL) and streptomycin (100 mg/mL) for 24 hours. Non‐adherent cells were collected as bone marrow‐derived macrophages (BMMs). BMMs were induced to OCPs (adherent cells) after treatment with macrophage colony‐stimulating factor (M‐CSF, 30 ng/mL in all experiments; Peprotech) for 3 days as previously described.^38^, ^39^ The cells were cultured in a humidified atmosphere containing 5% CO_2_ at 37°C. All media used were phenol red free to avoid oestrogen agonist effect, and serum (FBS, 12676029, Gibco) was stripped with charcoal to remove steroids.

### 2.3 Osteoclast differentiation assay

Osteoclast precursors (8 × 10^4^ cells/well) were cultured in 24‐well plates and treated with M‐CSF plus RANKL (100 ng/mL in all experiments; R&D Systems) for 5 days. Osteoclastogenesis was determined by tartrate‐resistant acid phosphatase (TRAP) staining with the relevant kit (Sigma‐Aldrich) according to the manufacturer's instructions. TRAP^+^ cells with more than 3 nuclei were considered differentiated osteoclasts. TRAP^+^ cells with more than 5 nuclei were considered large osteoclasts. Osteoclasts were counted in each well.

### 2.4 Determination of the experimental doses of 17β‐estradiol and 3‐MA

We employed two main reagents/drugs, 17β‐estradiol and 3‐MA (Sigma‐Aldrich), in this study. The range of in vitro doses for 17β‐estradiol treatment was selected based on previous studies.^18^, ^40^, ^41^, ^42^, ^43^ For the in vitro studies, we used a moderate concentration (5 nmol/L) for subsequent experiments based on the results of autophagic protein expression. For the in vivo studies, we selected a low dose (20 μg/kg, s.c. for 60 days) based on a previous investigation.^44^, ^45^ In addition, to determine the experimental dose of 3‐MA in vitro, we divided the OCPs into five groups (n = 3 per group), and each group received M‐CSF and RANKL with or without different concentrations of 3‐MA (0.5, 1, 2, 3, 5 mmol/L) for 5 days. Concentrations resulting in no difference in the number of mature osteoclasts from the control group were identified as the experimental doses of 3‐MA to be used. The experimental dose of 3‐MA in vivo was determined by comparing the recovery of bone loss between the control group and each experimental group (15, 30, and 50 mg/kg, i.p. for 60 days; n = 6, per group). Hence, 0.5 mmol/L of 3‐MA in vitro and 30 mg/kg of 3‐MA in vivo were used as experimental doses selected using the criteria described above.

### 2.5 Lentivirus transduction

Lentiviruses encoding Beclin1 gene (BECN1) and short hairpin RNA (shRNA) against Atg5, Atg7 or BECN1 (including the corresponding control vector) were constructed by homologous recombination between an expression vector (EX‐Puro‐Lv105) and cDNA/shRNA in 293 cells using a construction kit (GeneCopoeia) as previously described.^46^ After 2 days, the supernatants were collected, and the OCPs were incubated in the viral fluid containing 8 μg/mL polybrene at a MOI of 10 for 2 days. The infected cells were selected by puromycin (7.5 μg/mL). The expression of viral genes was observed using Western blot (WB) analyses.

### 2.6 WB analysis

Whole‐cell lysates from OCPs (approximately 2 × 10^6^ cells/well) with the indicated treatments were prepared from 6‐well plates. Lysates were packed into 10% SDS‐PAGE gels and transferred to polyvinylidene fluoride membranes (PVDF). Next, the PVDF membranes were incubated with antibodies against TRAF6 (1:1000; Santa Cruz Biotechnology, Inc), p‐ERK (1:1000), p‐JNK (1:1000), p‐P38 (1:1000), ERK (1:1000), JNK (1:1000), P38 (1:1000), Atg5 (1:1000), Atg7 (1:1000), Beclin1 (1:1000), LC3B (1:1000) and tubulin (Cell Signaling Technology). Horseradish peroxidase (HRP)‐linked secondary antibodies were used as the secondary antibodies. The bands were visualized using a chemiluminescence system (Amersham Imager 600; General Electric).

### 2.7 Coimmunoprecipitation assay

Preparation of the cell protein lysates and coimmunoprecipitation assays were performed as previously described.^47^ The antibodies used were RANK (IP, 1:100; IB, 1:1000) and TRAF6 (IP, 1:100; IB, 1:1000; Santa Cruz Biotechnology, Inc) antibodies.

### 2.8 Quantitative real‐time PCR analysis

Total RNA was extracted and purified by the TRIzol method. Synthesis of cDNA and real‐time quantitative PCR (qRT‐PCR) measurements were performed as described previously.^38^ The designed primer sequences for qRT‐PCR are shown in Table 1. qRT‐PCR was carried out using a SYBR Premix Ex TaqTM kit (TaKaRa) and an ABI7500 PCR system (Applied Biosystems, Thermo).

**TABLE 1:** Specific primer sequences for qPCR

Gene	Forward (5′‐3′)	Reverse (5′‐3′)
Atg5	ATGCGGTTGAGGCTCACTTTA	GGTTGATGGCCCAAAACTGG
Atg7	GTTCGCCCCCTTTAATAGTGC	TGAACTCCAACGTCAAGCGG
BECN1	CTAAGGCAGGCAGGAGGATG	GCTGGCCTC AAGAGATCCAT
Cyclophilin A	CGAGCTCTGAGCACTGGAGA	TGGCGTGTAAAGTCACCACC

### 2.9 Immunofluorescence assay

Cells (2 × 10^6^) were seeded on 6 cm dishes and stimulated with the indicated treatments. The treated OCPs were fixed using 4% paraformaldehyde (PFA). After perforation, the cells were blocked using 1% BSA and incubated with anti‐LC3B antibody (1:500; Cell Signaling Technology) at 4°C overnight. Next, the cells were stained with fluorochrome‐labelled secondary antibody for 30 minutes and then counterstained with DAPI for 10 minutes. Ultimately, the cells were observed and recorded via fluorescence microscopy (Olympus IX81). Cells with more than 5 LC3 puncta were defined as positive cells.^48^, ^49^

### 2.10 Cell proliferation analysis

Cell proliferation was assessed by CCK‐8 cell viability assays. For the CCK‐8 assay, 3000 of the indicated cells/well were seeded in 96‐well plates. After the indicated times (0, 1, 2, 3 and 4 days) of natural growth, the cells were collected, and CCK‐8 assays were carried out using the Cell Counting Kit‐8 in accordance with the manufacturer's protocol. The optical density at 450 nm (OD450) was measured using a Varioskan Flash reader (Thermo). Subsequently, the cell growth curves were drawn.

### 2.11 Ovariectomized mouse model

After several weeks of acclimatization, 10‐week‐old female mice were subjected to bilateral ovariectomies (OVX) under anaesthesia with ether. Three days later, the OVX mice were administered 3‐MA (30 mg/kg/d) by intraperitoneal injection and/or subcutaneous injection of 17β‐estradiol (20 μg/kg/d). After 60 days of treatment, the OVX mice were sacrificed. The tibiae were collected, wrapped in 0.9% saline‐soaked gauze and stored at −20°C.

### 2.12 Micro‐computed tomography

Three‐dimensional (3D) reconstructions of the cancellous bones in the proximal tibia metaphysis were made using micro‐computed tomography (micro‐CT, lCT‐80; Scanco Medical AG). The voxel size of micro‐CT was 10 μm. The parameters included BMD, bone volume density (BV/TV), trabecular number (Tb.N) and trabecular separation/spacing (Tb.Sp, n = 8, per group).

### 2.13 Haematoxylin and eosin, TRAP staining and immunofluorescence and immunohistochemistry analyses in tissues

The tibiae of the mice were fixed in 4% PFA for 48 hours and decalcified in 10% EDTA (pH 7.3) for 2 weeks at 4°C. All the samples were then dehydrated in a graded ethanol series and embedded in paraffin (Leica). The tibia sections (5 μm thick) were stained with haematoxylin and eosin (H&E) and the TRAP or alkaline phosphatase (ALP) staining kit (n = 8, per group). The trabecular bone area (%Tb.Ar) of the H&E‐stained sections was analysed by Image‐Pro Plus (IPP). The osteoclast number in the TRAP‐stained sections and osteoblast number in the ALP‐stained sections were measured using an eyepiece grid. For immunohistochemistry (IHC, OCN, 1:200; Abcam) or immunofluorescence (IF, LC3B, 1:500; Cell Signaling Technology; RANK, 1:500; Santa Cruz Biotechnology), the sections were incubated in citrate buffer (10 mmol/L citric acid, pH 6.0) overnight at 60°C to unmask the antigens. We incubated the primary antibodies overnight at 4°C and the secondary antibodies for 1 hour at room temperature (n = 6, per group). For IHC assays, the cytoplasm of positive cells in the trabecular area was stained red. The average integrated optical density of OCN^+^ cells was analysed by IPP as previously described.^50^ The LC3 fluorescence intensity of the RANK^+^ cells was analysed by IPP and calculated as the ratio of RANK^+^ LC3^+^ cells to RANK^+^ cells.

### 2.14 Detection of apoptosis

Apoptosis was evaluated by Annexin V–FITC/PI staining. After the treatment detailed in the experimental design, treated cells were collected, and then staining was performed according to the manufacturer's instructions. Subsequently, apoptotic cells were measured and quantitatively analysed using a flow cytometer (BD Accuri C6 Plus; BD Biosciences).

### 2.15 Osteoclastic bone resorption assay

Osteoclast precursors were stimulated with M‐CSF plus RANKL for 2 days, dissociated and reseeded onto sterilized bone discs (Corning) and were allowed to adhere to the surface for 6 hours before reagent treatment. The cells were then subjected to various treatments for an additional 6 days. Cells were removed by sonication and agitation, and the resorption pits were imaged using a scanning electron microscope (FEI Quanta 250; Thermo Fisher Scientific). Bone resorption areas were measured using ImageJ software.

### 2.16 Flow cytometry of isolated bone marrow RANK^+^ CD11b^+^ macrophages

Bone marrow cells were isolated from each group of OVX mice. Cells were dispersed into single‐cell suspensions using a 70 micron nylon mesh, washed and resuspended in 5 mL of DMEM on ice for 3 minutes. Non‐specific binding was blocked by pre‐treating cells with rat anti‐mouse CD16/32 monoclonal antibody (TruStain FcX™ PLUS; BioLegend) for 10 minutes at room temperature. Cells were stained using PE anti‐mouse CD265 (RANK) antibody and APC anti‐mouse CD11b antibody (BioLegend), and the cells were collected on a MoFlo XDP flow cytometer (Beckman Coulter). The collected cells were analysed for LC3 puncta formation using an IF assay.

### 2.17 Statistical analyses

Data are expressed as the mean ± SEM. Statistical analyses were carried out using one‐way ANOVA. The *P* value was set at .05. All statistical analyses were performed using SPSS 19.0 software.

## 3 RESULTS

### 3.1 Treatment with 17β‐estradiol enhanced the autophagic activity of the OCPs

**FIGURE 1:**
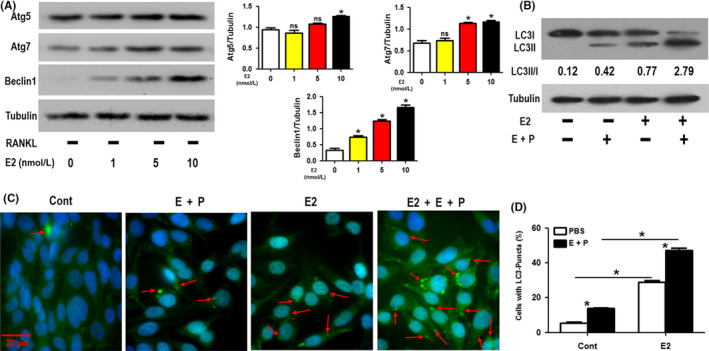
Treatment with 17β‐estradiol enhances the autophagic activity of OCPs. A, Following administration of different concentrations of 17β‐estradiol (0, 1, 5 or 10 nmol/L) for 8 h in the absence of RANKL, the Atg5, Atg7 and Beclin1 proteins in BMM‐derived OCPs were detected using Western blot analyses. B, The ratio of LC3II/I in the OCPs treated with 17β‐estradiol (5 nmol/L) for 8 h in the presence or absence of E64d plus PEPS A. C, After the OCPs were treated with the reagents as described for (B) for 12 h, LC3 puncta (red arrows) were imaged using immunofluorescence staining and then observed under fluorescence microscopy. Scale bar, 20 μm. D, Statistical diagram showing the percentages of cells with LC3 puncta in C (≥5 dots, 50 cells per field, n = 5). Data are expressed as the mean ± SEM from three independent experiments. *P < .05. Cont, control group; E, E64D; E2, 17β‐estradiol; ns, no significance; P, PEPS A

We first assessed the direct effect of 17β‐estradiol on the autophagic activity of the OCPs without administering RANKL. The Beclin1 protein level increased in a concentration‐dependent manner with the increasing 17β‐estradiol concentration in the OCPs (Figure 1A). We found that 17β‐estradiol improved Atg5 protein expression only at 10 nmol/L and Atg7 protein expression only at 5 and 10 nmol/L (Figure 1A). Notably, both the LC3 transformation (LC3II/I) and the formation of LC3 puncta in the OCPs increased significantly after treatment with 5 nmol/L 17β‐estradiol, and these parameters were further enhanced when the lysosomal protease inhibitors E64d and PEPS A were added (Figure 1B‐D). The results suggested that oestrogen could directly upregulate the autophagic activity of OCPs.

### 3.2 Treatment with 17β‐estradiol inhibited the RANKL‐induced autophagy of the OCPs

**FIGURE 2:**
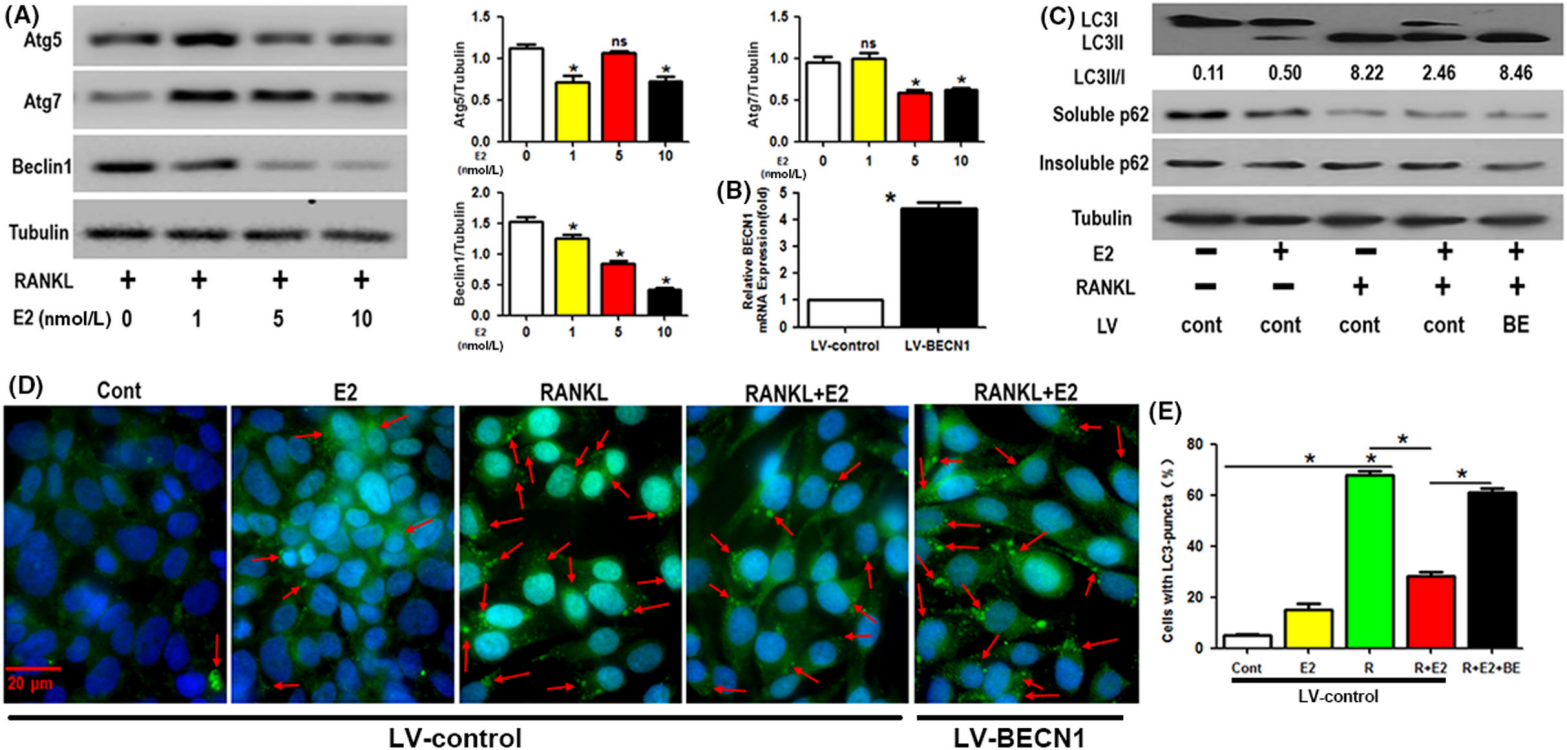
Treatment with 17β‐estradiol inhibits the RANKL‐induced autophagy of OCPs. A, Following administration of different concentrations of 17β‐estradiol (0, 1, 5 or 10 nmol/L) for 8 h in the presence of RANKL, the Atg5, Atg7 and Beclin1 proteins in OCPs were detected using Western blot analyses. B, The efficiency of lentivirus (LV‐BECN1) transduction was verified by detecting BECN1 mRNA level. C, The ratio of LC3II/I and the soluble p62, insoluble p62 proteins in the OCPs treated with 17β‐estradiol (5 nmol/L) and/or RANKL for 8 h after lentiviral transduction. D, Images of LC3 puncta (red arrows) in the OCPs treated with the above reagents for 12 h after lentiviral transduction. Scale bar, 20 μm. E, The percentages of cells with LC3 puncta in D. Data are expressed as the mean ± SEM from three independent experiments. *P < .05. BE, lentivirus encoding BECN1; Cont, control group; E2, 17β‐estradiol; ns, no significance; R, RANKL

We showed that 17β‐estradiol plays a direct role in promoting OCP autophagy. RANKL is also known to augment OCP autophagy^22^, ^51^; thus, we next studied the role of 17β‐estradiol in RANKL‐induced OCP autophagy. As shown in Figure 2A, 17β‐estradiol decreased the Beclin1 protein expression in a concentration‐dependent manner from 0 to 10 nmol/L. Treatment with 17β‐estradiol enhanced Atg5 protein expression only at 1 and 10 nmol/L and Atg7 protein expression only at 5 and 10 nmol/L (Figure 2A). Moreover, both 17β‐estradiol (5 nmol/L) and RANKL (100 ng/mL) enhanced the LC3 transformation and LC3 puncta formation (Figure 2C‐E). Unexpectedly, compared with the single RANKL group, the group treated with 17β‐estradiol and RANKL showed reductions in the above autophagic parameters (Figure 2C‐E). More interestingly, after stimulation by RANKL, overexpression of Beclin1 using lentivirus transduction (4.43‐fold) compensated for the 17β‐estradiol‐reduced LC3 transformation and LC3 puncta formation (Figure 2B,C‐E). Given that Beclin1 is a key downstream signal during RANKL‐induced autophagy of OCPs, our data indicated that oestrogen exerts an inhibitory effect on RANKL‐induced autophagy of the OCPs. Both 17β‐estradiol and RANKL inhibited the expression of soluble p62 while 17β‐estradiol reversed RANKL‐reduced soluble p62 protein level, which was recovered with Beclin1 expression (Figure 2C). However, insoluble p62 was stable among each group (Figure 2C). The above data supported the idea that autophagic flux was accelerated in our experimental system.

In addition, Annexin V–FITC/PI staining results showed that in the presence or absence of RANKL, the addition of 17β‐estradiol (5 nmol/L) promoted the apoptosis of OCPs (Figure S1A,B), which was similar to the previous results.^19^, ^20^ Furthermore, OCP apoptosis increased with RANKL treatment (Figure S1A,B), which was also consistent with previous research results showing that RANKL promoted OCP apoptosis at the beginning of the precursor stage.^52^, ^53^

### 3.3 Treatment with 17β‐estradiol suppressed the TRAF6‐MAPK signalling pathway downstream of RANKL

**FIGURE 3:**
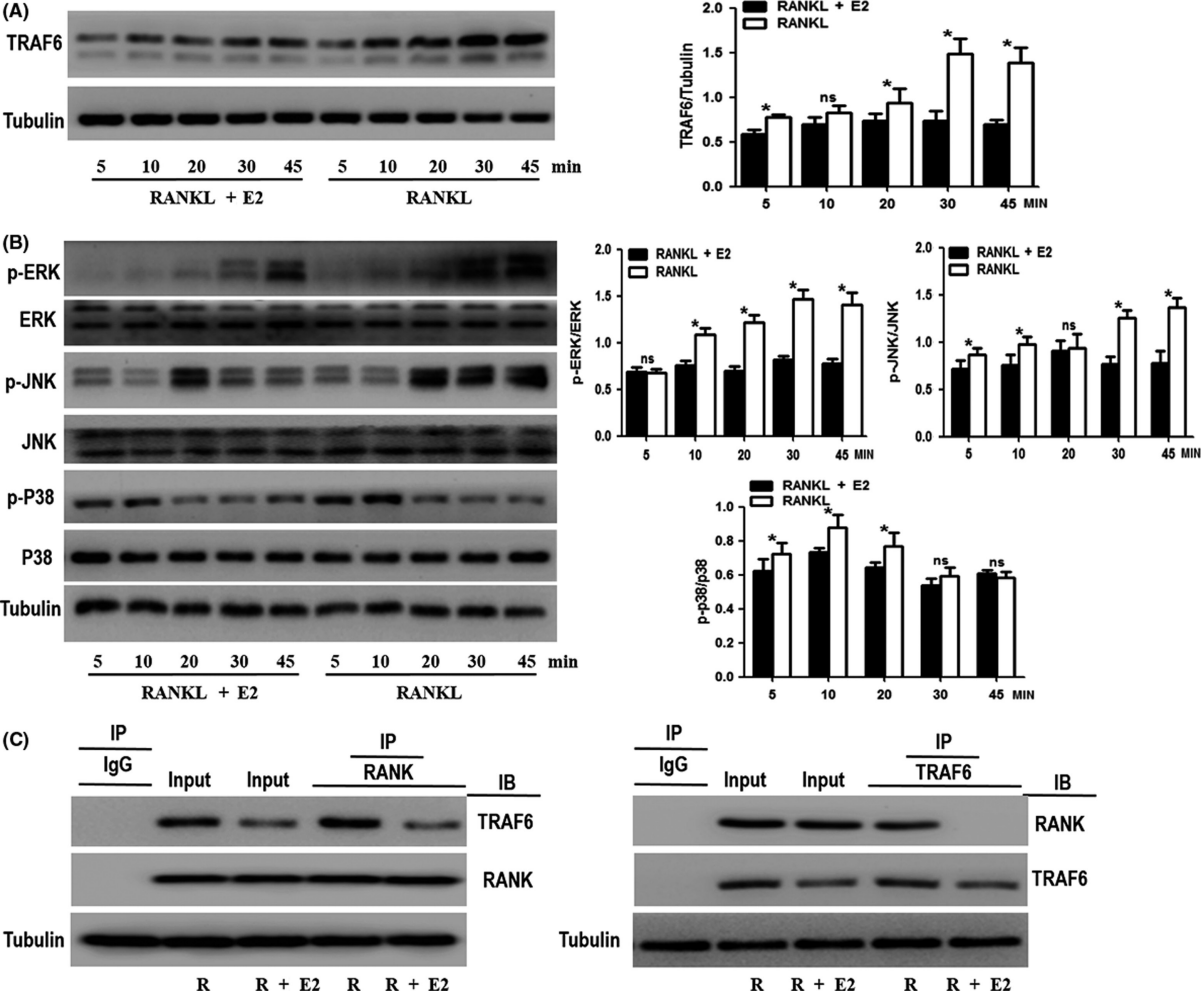
Treatment with 17β‐estradiol suppresses the TRAF6‐MAPK signalling pathway downstream of RANKL. A and B, OCPs were treated with or without RANKL for the indicated times. TRAF6, p‐ERK, p‐JNK and p‐P38 were detected using Western blot analyses. The histograms represent the comparisons of the expression levels of each protein between the RANKL‐lacking group and the RANKL group at the indicated time points. The expression of phosphorylated proteins is represented by the ratio of phosphorylated protein to total protein. C, OCPs were treated with or without RANKL for 2 h. Cell lysates were extracted for Co‐IP with anti‐RANK or anti‐TRAF6 antibody, and subsequently, precipitates were detected using Western blots with anti‐TRAF6 or anti‐RANK antibody, respectively. Data are expressed as the mean ± SEM from three independent experiments. *P < .05. E2, 17β‐estradiol; IB, the antibody for immunoblot; IP, the antibody for immunoprecipitation; ns, no significance; R, RANKL

It is known that 17β‐estradiol could inhibit RANKL‐induced autophagy in the OCPs. We speculate that the inhibitory effect is due to the 17β‐estradiol‐mediated inhibition of RANKL signalling. As shown in Figure 3A, after 17β‐estradiol was added, the TRAF6 protein expression was significantly decreased except at the 10 minutes time point. The MAPK signalling molecules in OCPs are activated downstream of RANK (RANKL receptor)/TRAF6 in RANKL‐induced osteoclastogenesis. Here, the activity changes of several key molecules related to the MAPK signalling pathway were investigated under 17β‐estradiol intervention. The expression of phosphorylated ERK (p‐ERK) decreased significantly except at the 5 minutes time point under 17β‐estradiol stimulation (Figure 3B). The expression of phosphorylated JNK (p‐JNK) was downregulated by 17β‐estradiol, except at 20 minutes (Figure 3B). The phosphorylated p38 (p‐p38) level was reduced by 17β‐estradiol, except at 30 and 45 minutes (Figure 3B). RANKL is known to induce the interaction of RANK and TRAF6. Co‐IP assays showed that the addition of 17β‐estradiol attenuated the coprecipitation level of RANK and TRAF6, indicating that 17β‐estradiol inhibited the interaction of RANK and TRAF6 (Figure 3C). The data above are the first evidence indicating that oestrogen represses TRAF6‐MAPK signalling downstream of RANKL.

### 3.4 Suppression of autophagic activity promoted the inhibitory effect of 17β‐estradiol on osteoclastogenesis

**FIGURE 4:**
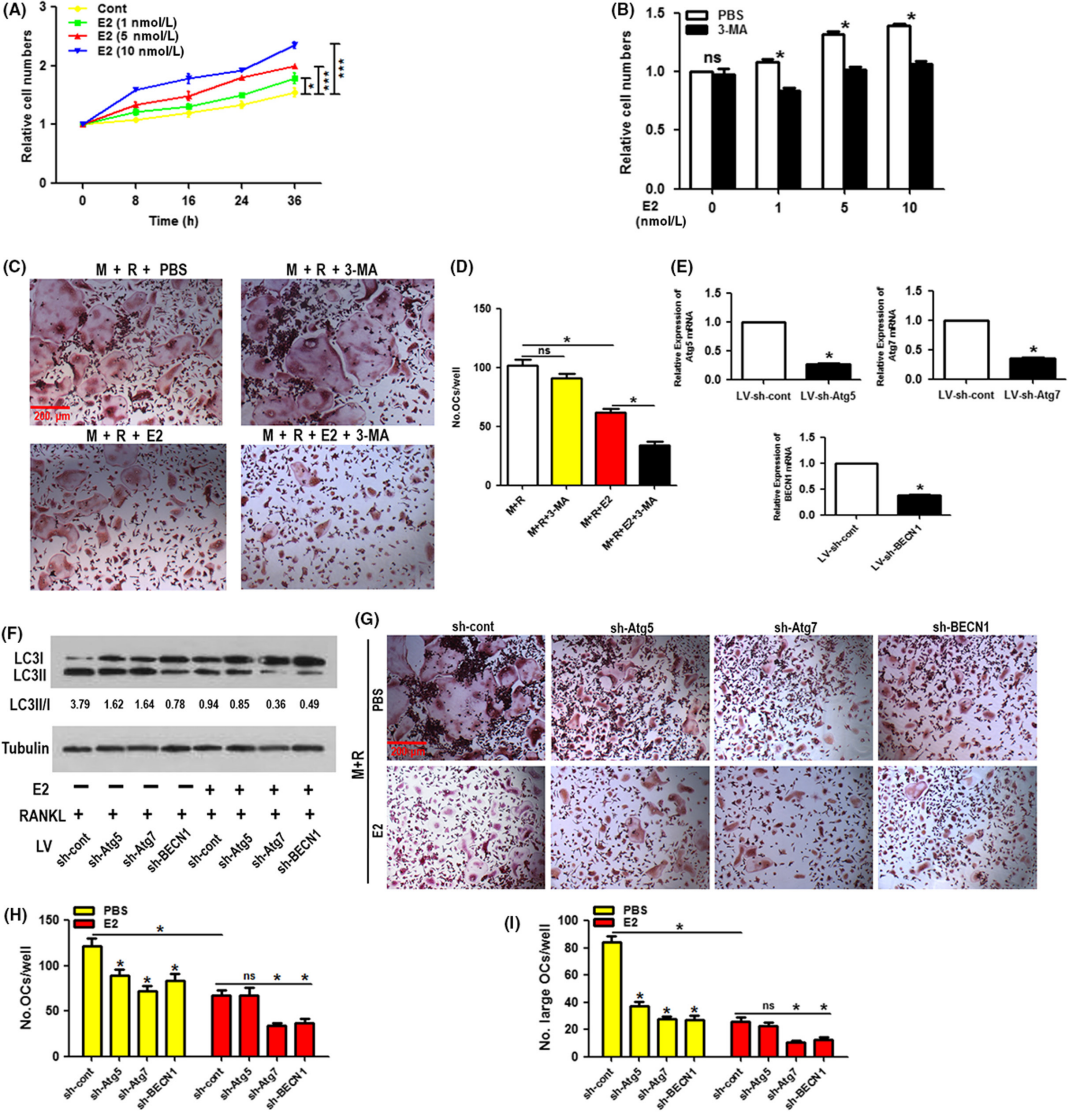
Suppression of autophagic activity promotes the inhibitory effect of 17β‐estradiol on osteoclastogenesis. A, The proliferation of the OCPs treated with 17β‐estradiol (0, 1, 5 or 10 nmol/L) for the indicated times was detected by CCK‐8 assays. B, The proliferation of the OCPs treated with 17β‐estradiol (0, 1, 5 or 10 nmol/L) along with PBS or 3‐MA (0.5 μmol/L) for 24 h was detected by CCK‐8 assays. C, The OCPs were treated with M‐CSF plus RANKL along with PBS, 3‐MA (0.5 μmol/L) and/or 17β‐estradiol (5 nmol/L) for 5 d. Representative images of TRAP‐positive multinucleated cells in each group. Scale bar, 200 μm. D, The quantitative results showed the number of TRAP‐positive multinucleated cells in C. E, The Atg5, Atg7 and BECN1 mRNA levels in the OCPs infected with lentiviruses encoding Atg5‐shRNA, Atg7‐shRNA, or BECN1‐shRNA (LV‐sh‐Atg5, LV‐sh‐Atg7, LV‐sh‐BECN1) or control viruses (LV‐sh‐cont). F, Following lentiviral transduction, the ratio of LC3II/I in the OCPs treated with 17β‐estradiol (5 nmol/L) in the presence of RANKL for 8 h was detected using Western blot analyses. G, Typical images of TRAP staining of the mature osteoclasts derived from the OCPs transduced with lentiviruses followed by M‐CSF plus RANKL along with PBS or 17β‐estradiol (5 nmol/L) treatment for 5 days. Scale bar, 200 μm. H and I, The quantitative results regarding mature osteoclasts (more than 3 nuclei) or large osteoclasts (more than 5 nuclei) in G. Data are expressed as the mean ± SEM from three independent experiments. *P < .05. E2, 17β‐estradiol; M, M‐CSF; ns, no significance; R, RANKL

Our data showed that while directly promoting OCP autophagy, 17β‐estradiol could also suppress RANKL‐induced OCP autophagy. Thus, the role of autophagy in 17β‐estradiol‐mediated anti‐osteoclastogenesis requires further investigation. To achieve this goal, we employed the autophagic inhibitor 3‐MA. After administration of different levels of 17β‐estradiol for the indicated times, we found that cell growth was elevated in the absence of RANKL, and the most effective concentration of 17β‐estradiol was 10 nmol/L (Figure 4A). However, the effects of 17β‐estradiol were reduced by 0.5 mmol/L of 3‐MA for 24 hours (Figure 4B). We also observed that 17β‐estradiol (5 nmol/L) reduced the number of differentiated osteoclasts induced by RANKL (50 ng/mL) and M‐CSF (30 ng/mL; Figure 4C,D). More importantly, 3‐MA alone had no effect on the level of mature osteoclasts but enhanced the effect of 17β‐estradiol when added (Figure 4C,D). Upon treatment with the same concentration of reagents, the osteoclastic bone resorption assay showed similar results as the above‐mentioned osteoclast differentiation experiments (Figure S2A,B), indicating that 3‐MA also enhanced the inhibition of 17β‐estradiol on osteoclastic function.

Subsequently, a gene silencing technique was employed to further explore which autophagic gene was involved in 17β‐estradiol‐regulated osteoclastogenesis. As shown in Figure 4E‐I, in the presence of RANKL and M‐CSF, shRNA‐mediated knock‐down of Atg7 or BECN1 (0.35‐fold; 0.38‐fold) not only reduced osteoclast numbers (including the number of large osteoclasts) and the LC3 transformation in OCPs but also enhanced the inhibitory effect of 17β‐estradiol (5 nmol/L) on mature osteoclasts (including large osteoclasts) as well as the LC3 transformation in OCPs. Nevertheless, although Atg5 silencing (0.26‐fold) reduced osteoclast differentiation and the LC3 transformation in OCPs, it exerted no additional effects on the function of 17β‐estradiol (Figure 4E‐I). Thus, we inferred that Atg7 or Beclin1‐involved autophagic activation may play a role in 17β‐estradiol‐regulated osteoclastogenesis. These results are the first evidence showing that 17β‐estradiol directly improved OCP proliferation levels while suppressing RANKL‐induced osteoclast differentiation and support the hypothesis that autophagic inhibition further suppresses 17β‐estradiol‐regulated osteoclastogenesis.

### 3.5 Treatment with 3‐MA increases the inhibitory effect of 17β‐estradiol on LC3 fluorescence intensity in trabecular OCPs in OVX mice

**FIGURE 5:**
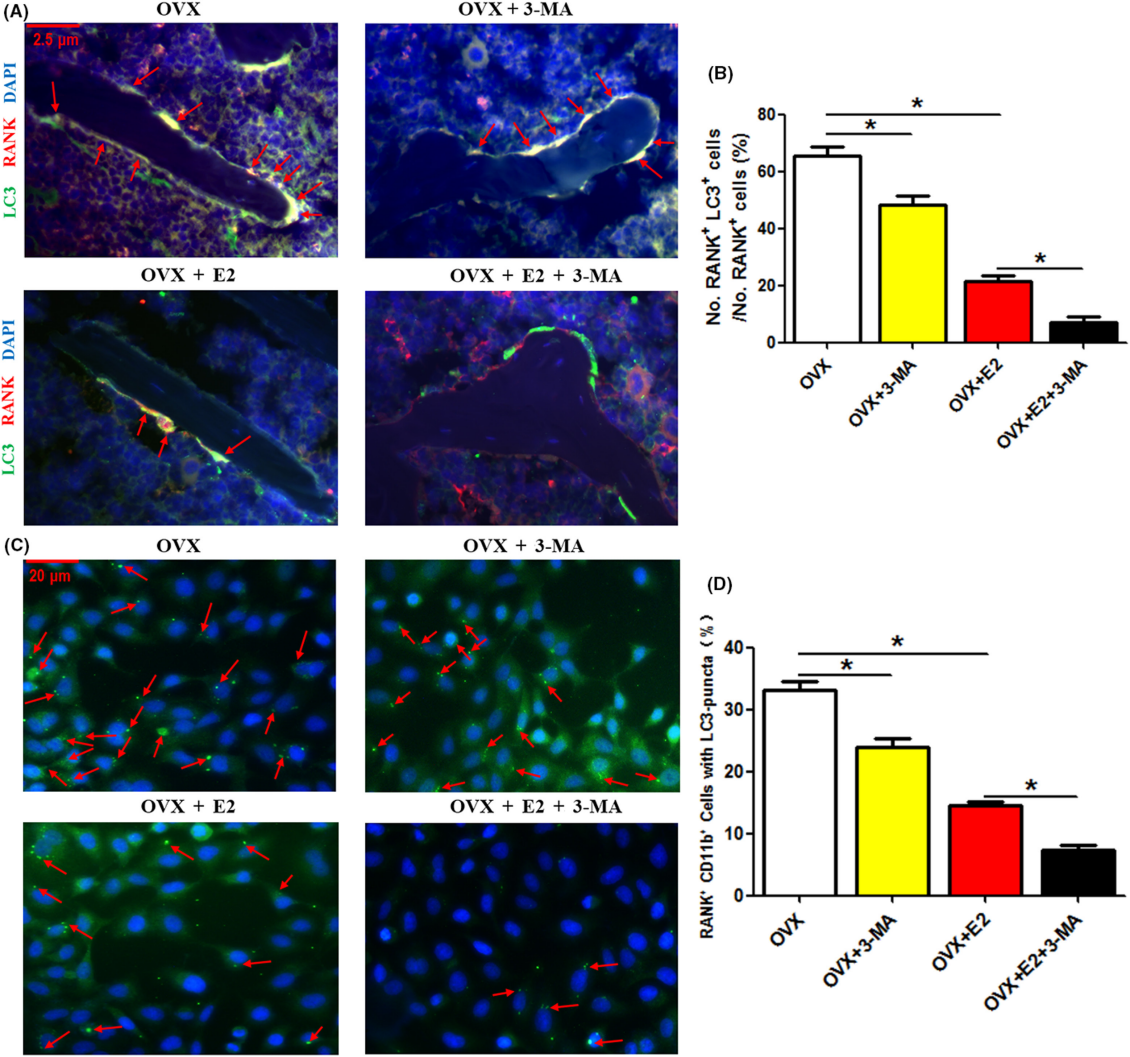
Treatment with 3‐MA increases the inhibitory effect of 17β‐estradiol on LC3 fluorescence intensity in trabecular OCPs in OVX mice. The OVX‐operated 10‐wk‐old female mice were treated with PBS or 3‐MA (30 mg/kg, i.p. for 60 d) and/or 17β‐estradiol (20 μg/kg, s.c. for 60 d). A, Tibial sections were stained with green and red fluorochromes for LC3B and RANK, respectively, and observed using fluorescence microscopy. The overlapping staining of LC3B and RANK are indicated with red arrows (yellow fluorescence). Scale bar, 2.5 μm. B, Statistical diagram indicates the quantitative results of IF assays (the ratio of RANK^+^ LC3^+^ cells/RANK^+^ cells; 100 cells per mouse were counted). C, Images of LC3 puncta (red arrows) in bone marrow RANK^+^ CD11b^+^ cells sorted by flow cytometry. D, The percentages of cells with LC3 puncta in C (N = 3). E2, 17β‐estradiol; OVX, ovariectomized mice

Given the in vitro effect of 17β‐estradiol on upregulating autophagy in OCPs, autophagic inhibition could further reduce 17β‐estradiol‐regulated autophagy of OCPs. Accordingly, the relationship between 17β‐estradiol and autophagy in vivo should be further explored. We used RANK (specific marker of OCPs attached to bone matrix)‐positive cells in vivo to indicate trabecular bone OCPs in OVX mice (osteoporosis model). Immunofluorescence double staining showed that not only 3‐MA (30 mg/kg, i.p. for 60 days) but also 17β‐estradiol (20 μg/kg, s.c. for 60 days) significantly reduced the LC3 fluorescence intensity of the RANK^+^ cells in the bone trabecula of OVX mice (Figure 5A,B). More importantly, the addition of 3‐MA further decreased the LC3 fluorescence intensity of the RANK^+^ cells inhibited by 17β‐estradiol (Figure 5A,B), implying that autophagy in the trabecular bone OCPs inhibited by 17β‐estradiol still has space to further reduce. After isolating the RANK^+^ CD11b^+^ bone marrow macrophages (regarded as differentiated OCPs), it was found that the trend of LC3 puncta formation in the isolated cells of each group of OVX mice is consistent with that of LC3 fluorescence intensity in trabecular bone OCPs (Figure 5C,D), supporting the above observation.

### 3.6 Treatment with 3‐MA enhanced the ability of 17β‐estradiol to ameliorate bone loss and decrease osteoclastogenesis in OVX mice

**FIGURE 6:**
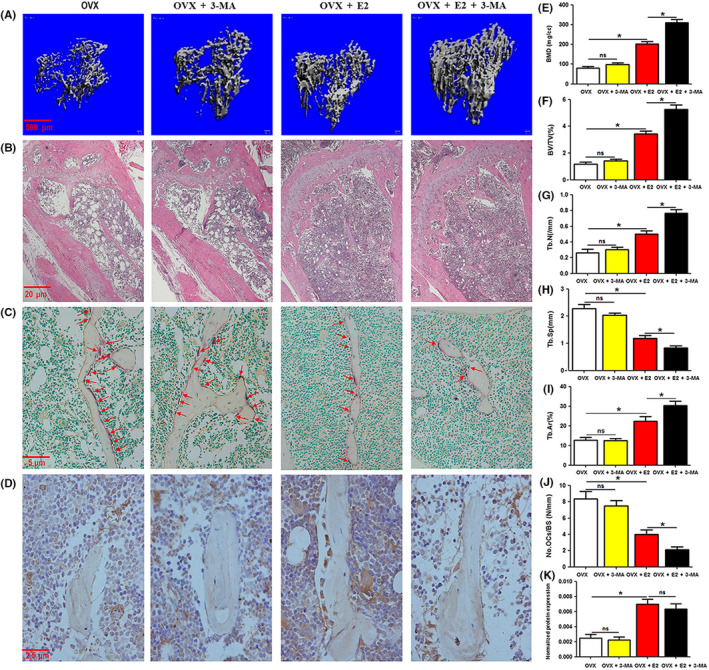
Treatment with 3‐MA enhances the ability of 17β‐estradiol to ameliorate bone loss and decrease osteoclastogenesis in OVX mice. The OVX‐operated 10‐wk‐old female mice were treated with PBS or 3‐MA (30 mg/kg, i.p. for 60 d) and/or 17β‐estradiol (20 μg/kg, s.c. for 60 d). A, Representative 3D micro‐CT reconstructed images of the tibiae from each group. Scale bar, 100 μm. B, Representative H&E‐stained tibial sections from each group. Scale bar, 20 μm. C, Representative TRAP‐stained tibial sections from each group (red arrows indicate TRAP^+^ cells). Scale bar, 5 μm. D, Representative IHC‐stained tibial sections of OCN from each group. E‐H, The trabecular bone parameters, including BMD, BV/TV, Tb.N and Tb.Sp, were analysed using micro‐CT (N = 8). I, The trabecular bone parameter, Tb.Ar, was analysed by H&E staining and using the IPP system (N = 8). J, The number of osteoclasts per millimetre of trabecular bone surface was counted (N = 8). K, Statistical diagram indicates the quantitative results of IHC assays (N = 6). Data are expressed as the mean ± SEM. *P < .05. E2, 17β‐estradiol; ns, no significance; OVX, ovariectomized mice

We showed that autophagic inhibition further abated the osteoclastogenesis regulated by 17β‐estradiol. Furthermore, in vivo and in vitro experiments showed that the autophagy of OCPs treated with 17β‐estradiol was further decreased by autophagic inhibition. Next, we evaluated the in vivo role of autophagy in 17β‐estradiol‐regulated bone loss using OVX mice. The low dose of 17β‐estradiol was employed for the in vivo study. Micro‐CT results showed that trabecular bone loss in OVX mice was ameliorated by 17β‐estradiol treatment, and this effect was further enhanced by coadministration with 3‐MA (Figure 6A). Bone parameter analysis of the tibiae showed that the administration of 17β‐estradiol efficiently increased BMD, BV/TV and Tb.N and decreased Tb.Sp in OVX mice, whereas the combination of 17β‐estradiol and 3‐MA had a stronger effect than 17β‐estradiol monotherapy (Figure 6E‐H). Likewise, H&E staining showed that the trabecular areas in OVX mice were increased by 17β‐estradiol and further ameliorated by coadministration of 17β‐estradiol and 3‐MA (Figure 6B,I). However, the 3‐MA monotherapy had no significant effect on the above bone parameters (Figure 6A,B,E‐I). Therefore, we confirmed that an autophagic inhibitor could enhance the protective effect of 17β‐estradiol.

More importantly, we found that the TRAP‐positive cells in the bone trabecula of OVX mice were significantly reduced by 17β‐estradiol administration and decreased further following the coinjection of 3‐MA (Figure 6C,J). This result indicated that the suppression of osteoclastogenesis might contribute to the protective effect of 17β‐estradiol and 3‐MA coadministration against bone loss. In addition, IHC assays showed that although 17β‐estradiol administration increased the expression level of osteocalcin (OCN, an in vivo osteoblastic marker), 3‐MA failed to promote its effect (Figure 6D,K). ALP staining (osteoblast‐specific staining) demonstrated similar results to the OCN IHC staining (Figure S3A,B). These results indicated that as an autophagic inhibitor, 3‐MA could improve the efficacy of 17β‐estradiol in preventing bone loss by in vivo inhibition of osteoclastogenesis.

## 4 DISCUSSION

Oestrogen has been shown to have a negative effect on osteoclastogenesis^16^, ^17^, ^18^, ^19^, ^20^ and is widely accepted as an autophagic activator.^25^, ^26^, ^27^, ^28^, ^29^, ^30^, ^31^, ^32^ An intriguing viewpoint is that autophagy facilitates osteoclastogenesis. Thus, there might exist a counteracting mechanism between osteoclastogenesis and the autophagic activity of OCPs after oestrogen treatment, which should be further investigated. Here, we first clarified the discrepant role of 17β‐estradiol in the autophagic response of OCPs with or without RANKL. Data from our in vitro experiment showed that 17β‐estradiol at concentrations from 1 to 10 nmol/L elevated autophagy‐related proteins and a concentration of 5 nmol/L increased the LC3 transformation and LC3 puncta formation. However, 17β‐estradiol (5 nmol/L) exerted an inhibitory effect on RANKL‐induced autophagy of the OCPs, which was reversed by Beclin1 overexpression. This finding might be caused by the decreased RANKL efficiency after oestrogen administration. Streicher and Liang et al^54^, ^55^ showed that oestrogen suppressed osteoclastogenesis by reducing the expression of RANKL. Chen and Robinson et al^40^, ^41^ found significant inhibition of RANKL‐induced osteoclast differentiation following oestrogen treatment. Oestrogen could also inhibit osteoclast formation through suppression of the RANKL‐induced downstream signalling pathway.^41^, ^42^, ^43^ In the presence of RANKL, its receptor, RANK, binds to TRAF6, subsequently triggering downstream signalling cascades, among which MAPK signalling is the most representative. Accordingly, RANKL‐induced TRAF6/MAPK signalling could be used to verify the effect of oestrogen on RANKL. The experimental results showed that oestrogen not only inhibited TRAF6 protein expression and the activity of MAPK signalling molecules treated with RANKL but also prevented the interaction of RANK and TRAF6. Therefore, we further confirmed the inhibitory effect of oestrogen on RANKL signalling in this study, which elucidated the inhibitory effect of oestrogen on RANKL‐induced autophagy of the OCPs. In summary, although oestrogen has a direct pro‐autophagy effect, it could block RANKL‐induced autophagic activation in OCPs by inhibiting RANKL signalling, exceeding the direct promotion of oestrogen on autophagy; thus, oestrogen exhibits a net anti‐osteoclastogenic effect. Nevertheless, the concomitant autophagic activation may exert a negative effect on the anti‐osteoclastogenic effect of oestrogen. Combined with the corresponding biological analyses, our findings confirmed the above hypothesis. In addition, it was observed that oestrogen promoted both apoptosis and autophagy in OCPs without RANKL. Combined with the results of Figure 3A,B, these findings suggested that oestrogen‐enhanced autophagy may promote OCP proliferation but not inhibit OCP apoptosis. These results suggest that oestrogen‐induced OCP apoptosis may not be mediated by autophagy alteration. However, under the direct intervention of oestrogen, the potential relationship between autophagy and apoptosis in OCPs needs further exploration. Nevertheless, in the presence of RANKL, oestrogen inhibited OCP autophagy and promoted OCP apoptosis. It was indicated that during RANKL‐induced osteoclastogenesis, the inhibitory effect of oestrogen on RANKL‐induced OCP autophagy could promote apoptosis. This observation was also verified by the finding that autophagy upregulation with BECN1 overexpression promoted the apoptosis of OCPs.

Consistent with previous studies,^16^, ^17^, ^18^, ^40^, ^41^, ^42^, ^43^ our results showed that 17β‐estradiol (5 nmol/L) significantly attenuated osteoclastogenesis and osteoclastic function. However, 17β‐estradiol unexpectedly promoted OCP proliferation at concentrations from 1 to 10 nmol/L. We hypothesized that the aberrant effect of oestrogen on OCP proliferation was due to its direct promoting effect on autophagy of the OCPs, which was verified by the inhibition of autophagy with 3‐MA. Importantly, 3‐MA not only reversed the promotion of OCP proliferation by 17β‐estradiol but also further enhanced the inhibitory effect of 17β‐estradiol on the formation and function of osteoclasts. Therefore, although oestrogen plays an anti‐osteoclastogenic role, it could directly stimulate the autophagy of OCPs, resulting in partial positive regulation of osteoclastogenesis, which counteracts its function. This inference was also proven by gene silencing techniques. Our experimental results demonstrated that the effects of 17β‐estradiol (5 nmol/L) on autophagy and osteoclastogenesis of the OCPs were enhanced by Atg7 silencing or BECN1 silencing. Notably, similar results were not obtained with Atg5 silencing. Accordingly, the role of different autophagy genes in oestrogen‐regulated autophagy and osteoclastogenesis of the OCPs requires further study. Likewise, the in vivo study also revealed that 3‐MA (30 mg/kg/d for 60 days) not only augmented the inhibition of 17β‐estradiol (20 μg/kg/d for 60 days) on the LC3 fluorescence intensity of the trabecular or bone marrow OCPs in OVX mice but also enhanced the protection of 17β‐estradiol against bone loss, which was mediated by the reduced osteoclastogenesis. However, both the in vitro and in vivo experiments showed that the low dose of 3‐MA exerted no individual effect on osteoclastogenesis and bone loss when enhancing the efficiency of 17β‐estradiol, which further verified that the synergistic action between oestrogen and autophagic inhibition was due to oestrogen‐activated autophagy. Notably, 3‐MA had no further effect on the 17β‐estradiol‐promoted OCN level and ALP‐positive cells of the OVX mice, which emphasizes the specificity of 3‐MA in regulating osteoclastogenesis.

**FIGURE 7:**
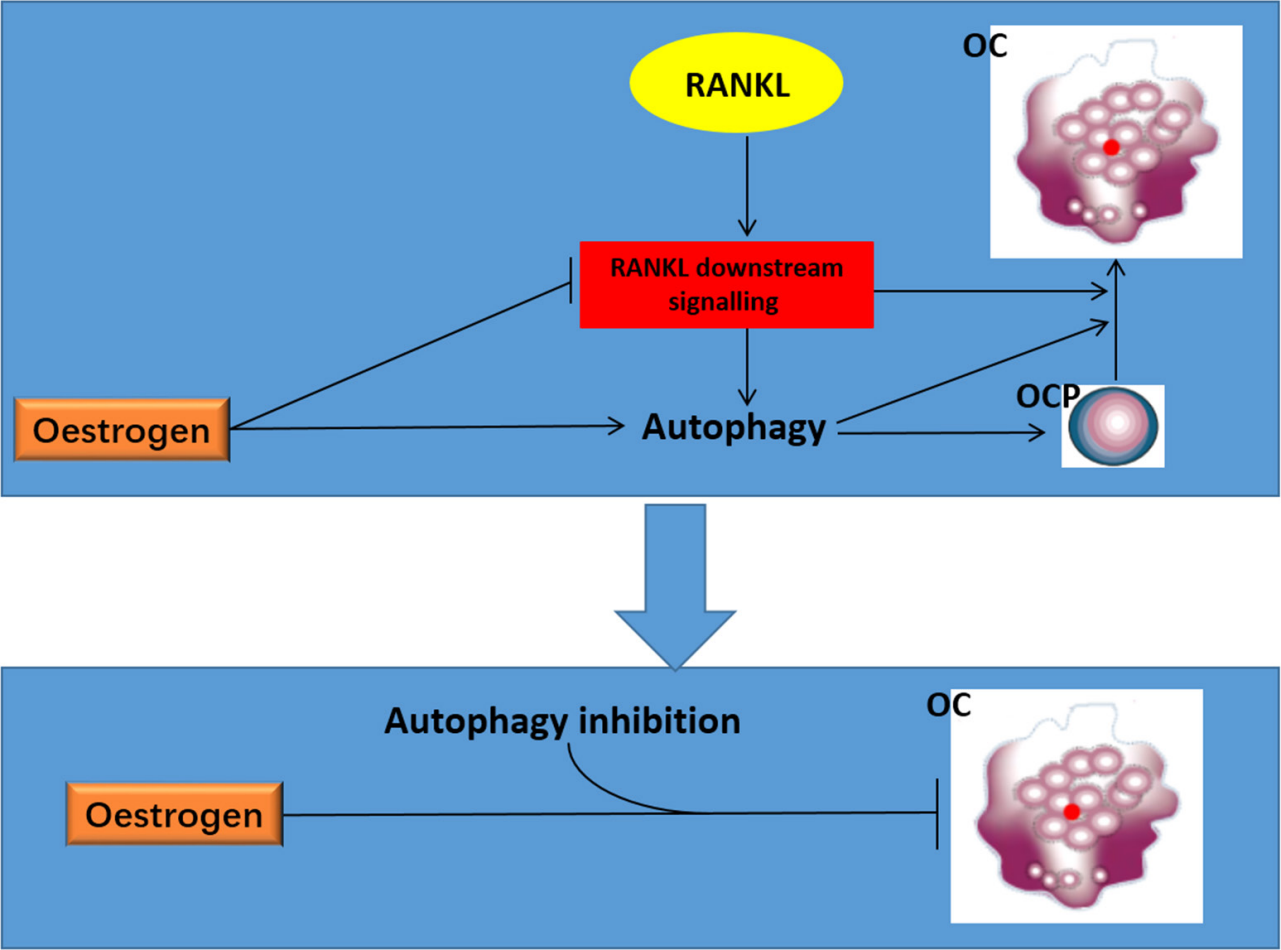
The working model regarding oestrogen‐regulated autophagy during osteoclastogenesis. In brief, autophagy plays a significant role in the formation of mature osteoclasts derived from OCPs. Oestrogen directly activates autophagy in OCPs, which contributes to OCP proliferation. However, oestrogen inhibits osteoclast differentiation by inhibiting RANKL downstream signalling, in which the inhibition of autophagy activated by RANKL signalling is also an important pathway. Accordingly, autophagy inhibition enhances the inhibitory effect of oestrogen on osteoclastogenesis. OC, osteoclast; OCP, osteoclast precursor; 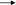 promote; 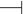 inhibit; 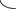 synergy

Oestrogen is considered an autophagic activator and osteoclastogenesis inhibitor. However, the intrinsic mechanism underlying this paradoxical phenomenon is unclear. We provide the first evidence showing that oestrogen can directly activate autophagy of OCPs and inhibit RANKL‐induced autophagy of the OCPs by downregulating RANKL signalling. More interestingly, suppression of autophagic activity further enhances the inhibitory effect of oestrogen on osteoclastogenesis. The working model regarding oestrogen‐regulated autophagy during osteoclastogenesis is shown in Figure 7. Thus, this study not only explores the underlying mechanism regarding the paradoxical role of oestrogen on OCP autophagy and osteoclastogenesis but also presents potential clues for the improvement of clinical strategies utilizing oestrogen in the treatment of osteoporosis.

## CONFLICT OF INTEREST

The authors have no competing interests to disclose.

## AUTHOR CONTRIBUTIONS

D.K. and D.X. conceived and designed experiments; D.Ke., L.C. and Y.Z. performed experiments, analysed data and prepared figures; D.K. and D.X. wrote the manuscript; D.X. provided technical expertise and edited the article.

## Supporting information

Figure S1‐S3

## Data Availability

All data generated or analysed during this study are included in this published article. And all data used to support the findings of this study are available from the corresponding author upon reasonable request.
