# Correction to “Oestrogen‐activated autophagy has a negative effect on the anti‐osteoclastogenic function of oestrogen”

**DOI:** 10.1111/cpr.13571

**Published:** 2024-01-03

Cheng L, Zhu Y, Xie D, Ke D. Oestrogen‐activated autophagy has a negative effect on the anti‐osteoclastogenic function of oestrogen. *Cell Prolif*. 2020;53:e12789. doi:10.1111/cpr.12789

The order of appearance of the third and fourth authors in the author list has been transposed.

The original author list is as follows:

Liang Cheng, Yunrong Zhu, Dianshan Ke, Denghui Xie

The corrected author list is as follows:

Liang Cheng, Yunrong Zhu, Denghui Xie, Dianshan Ke

The online version of this article was corrected.

We apologize for this error.

