# Supplementary material for: Oestrogen‐activated autophagy has a negative effect on the anti‐osteoclastogenic function of oestrogen
Source: Cell Prolif. 2020 Mar 11;53(4):e12789. doi: 10.1111/cpr.12789 (PMC7162800; doi:10.1111/cpr.12789)
Supplement: Supplementary file 1 — Figure S1‐S3 [file CPR-53-e12789-s001.docx]

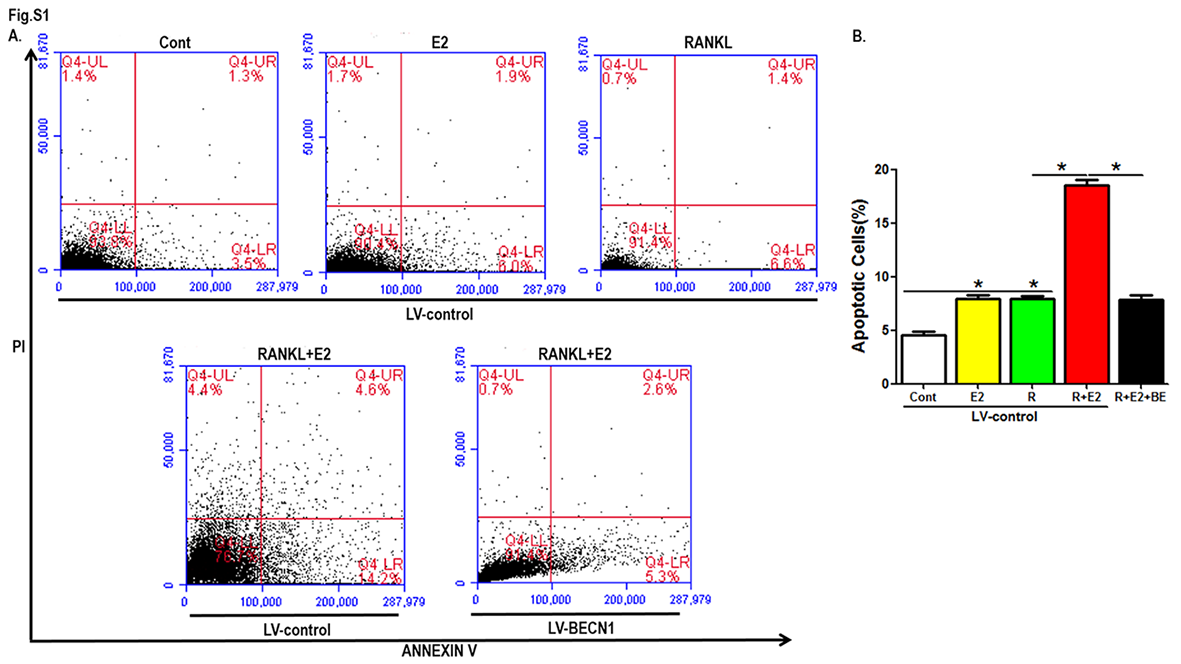


Fig. S1. 17β-estradiol promotes the apoptosis of OCPs in the presence or absence of RANKL. (A) After lentivirus transduction (LV-control or LV-BECN1), OCPs were treated with 17β-estradiol (5 nM) and/or RANKL for 12 hours, and then cell apoptosis was examined by flow cytometry detecting Annexin V-FITC/PI staining. (B) The percentages of apoptotic cells (ANNEXIN^+^ represents all apoptotic cells) are shown in statistical diagrams according to the results in A. Data are expressed as the mean±SEM from three independent experiments. *P<0.05. E2, 17β-estradiol; R, RANKL; Cont, control group; BE, lentivirus encoding BECN1.

**
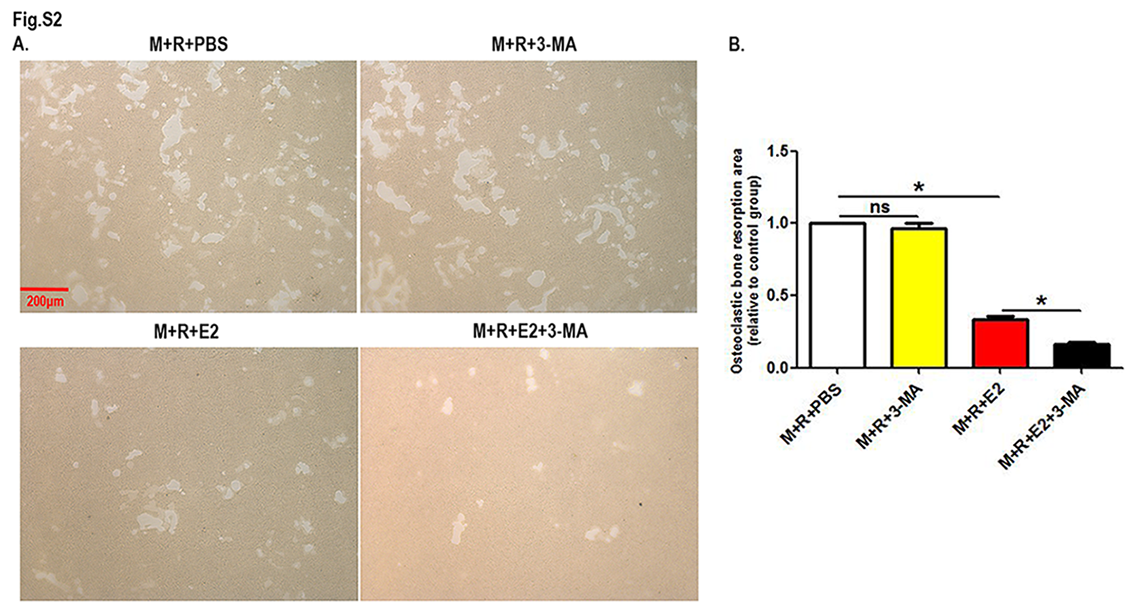
**

Fig. S2. Suppression of autophagic activity promotes the inhibitory effect of 17β-estradiol on osteoclastic bone resorption. (A) The bone resorptive activity of osteoclasts derived from OCPs treated with M-CSF plus RANKL along with PBS, 3-MA (0.5 μM) and/or 17β-estradiol (5 nM) for 6 days were assessed by scanning electron microscopy. Scale bar, 200 μm. (B) The quantitative results showed the mean resorption pit area in A. The resorption pit area was represented as normalized to that of control samples (M+R+PBS Group). Data are expressed as the mean±SEM from three independent experiments. *P<0.05. ns, no significance; E2, 17β-estradiol; R, RANKL; M, M-CSF.

**
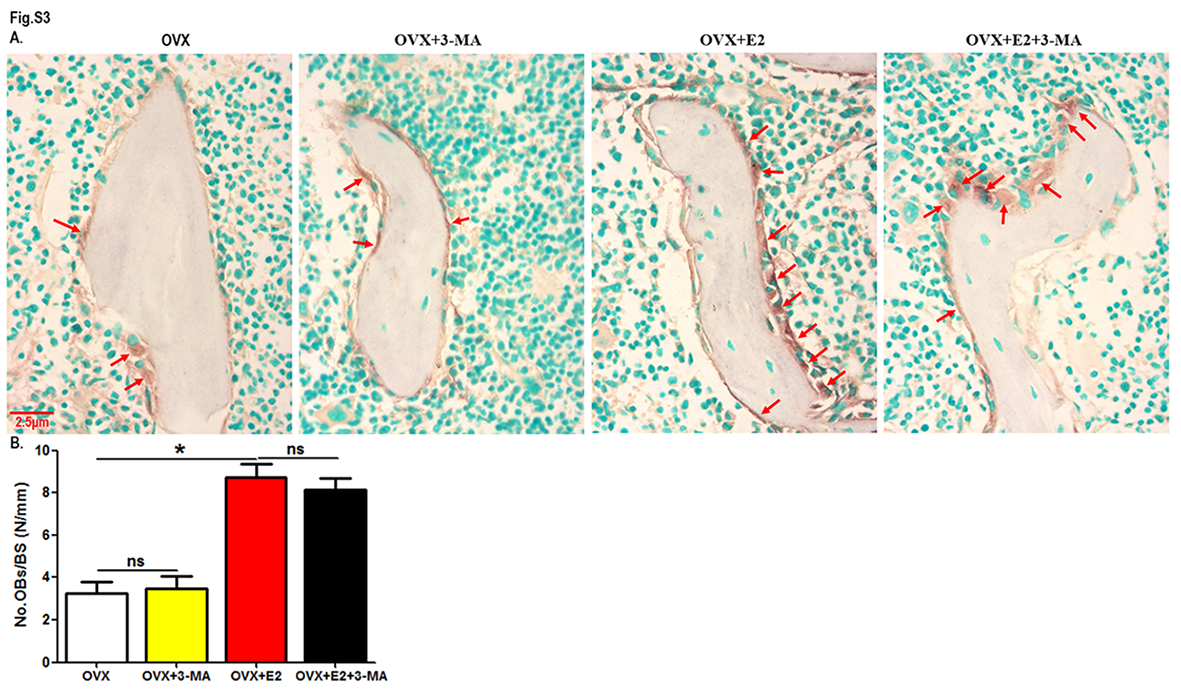
**

Fig. S3. Treatment with 3-MA does not affect the ability of 17β-estradiol to increase ALP-positive cells in the trabecular bone of OVX mice. The OVX-operated 10-week-old female mice were treated with PBS or 3-MA (30 mg/kg, i.p. for 60 days) and/or 17β-estradiol (20 μg/kg, s.c. for 60 days). (A) Representative ALP-stained tibial sections from each group (Red arrows indicate ALP^+^ cells). Scale bar, 2.5 μm. (B) The number of osteoblasts per millimeter of trabecular bone surface was counted (N=8). Data are expressed as the mean±SEM. *P<0.05. ns, no significance; OVX, Ovariectomized mice; E2, 17β-estradiol.
